# Association of serum uric acid Levels with metabolic syndromes in Korean adolescents

**DOI:** 10.3389/fendo.2023.1159248

**Published:** 2023-12-19

**Authors:** Young-Jun Seo, Young Suk Shim, Hae Sang Lee, Jin Soon Hwang

**Affiliations:** ^1^ Department of Pediatrics, Hallym University Chuncheon Sacred Heart Hospital, Chuncheon, Gangwon-do, Republic of Korea; ^2^ Department of Pediatrics, Ajou University Hospital, Ajou University School of Medicine, Suwon, Gyeonggi-do, Republic of Korea

**Keywords:** serum uric acid, children, adolescents, metabolic syndrome, reference values, cardiometabolic risks

## Abstract

**Introduction:**

The study findings investigated uric acid reference values and their association with a cluster of cardiometabolic risk factors among adolescents using the Korea National Health and Nutrition Examination Survey (KNHANES).

**Methods:**

A retrospective cross-sectional study was conducted using the KNHANES database from 2016 to 2018, involving a total of 2,462 participants aged between 10 and 18 years. Based on age- and sex-specific percentile curves for serum uric acid (SUA) levels from the KNHANES, we examined the correlation between cardiometabolic risk factors and serum uric acid levels.

**Results:**

The percentile values of SUA varied with sex and age. In male subjects, SUA levels tended to increase from 10 to 14 years of age and plateaued after 14 years of age. Moreover, the overall uric acid level in females was found to be lower than that in males; the levels tended to increase at approximately 10 to 12 years old but were relatively consistent according to age. Mean uric acid levels increased according to obesity status in both males and females. However, correlation analysis revealed that SUA levels were associated with several metabolic risks even after adjusting for obesity. The detailed metabolic syndrome (MetS) components that were observed to be associated with an increase in uric acid levels were different between males and females, but overall, high uric acid levels increased MetS risk. Additionally, a significant increase in MetS-related odds ratio (OR) for components, including waist circumference (WC), triglyceride (TG) levels, and low high-density lipoprotein cholesterol (HDL-c), was observed. However, differences between sexes were apparent, with a more pronounced increase in OR based on SUA levels in girls.

**Discussion:**

SUA levels were closely associated with MetS and its components, even in nonobese subjects. Therefore, high SUA levels in children and young adolescents should be closely monitored to prevent MetS.

## Introduction

Metabolic syndrome (MetS) is a clinical diagnosis characterized by a group of physiological, biochemical, and clinical risk factors that often occur concurrently, including central obesity, dysregulation of glucose homeostasis, dyslipidemia such as hypertriglyceridemia or low high-density lipoprotein cholesterol (HDL-c), and hypertension (1). Although there is still no clear consensus on the diagnostic criteria for pediatric MetS (2), the global prevalence of MetS was estimated to be 2.8% in children and 4.8% in adolescents in 2020 (3). In Korean population studies, the current prevalence was estimated to be between 2.2% and 6.2% based on the 2016-2018 Korea National Health and Nutrition Examination Survey (KNHANES); that trend has been increasing compared to the last decade (4–6). Overweight and obesity have been implicated as significant risk factors for the development of MetS in children and adolescents (7, 8). Meanwhile, the global prevalence of obesity in male and female children aged 5–19 years dramatically increased from 0.7% and 0.9% in 1975 to 5.6% and 7.8%, respectively, in 2016 (9). Therefore, efforts to treat and prevent MetS in children and adolescents are being emphasized worldwide; early screening, early diagnosis, and proper management are generally accepted as important factors for reducing the risk of cardiovascular diseases (10).

Uric acid is the catabolic byproduct of purine metabolism and is produced in the liver and excreted by the kidney. Serum uric acid (SUA) has been considered to have both beneficial and detrimental roles in the body depending on its concentration. On the one hand, uric acid has antioxidant properties under conditions of oxidative stress, accounting for more than 60% of the antioxidant capacity of blood plasma (11). However, hyperuricemia can lead to oxidative stress through the formation of radicals in reactions with other oxidant molecules, as an adaptive response (12), which also results in the precipitation of monosodium urate crystals. It is widely known that excess body fat can lead to insulin resistance, which can also increase uric acid production (13) and a decrease in uric acid excretion (14). Finally, elevated uric acid levels impair endothelial function and aggravate insulin resistance consequently increasing cardiovascular disease risk (15). Although various clinical and epidemiological studies have suggested a relationship between the uric acid level or hyperuricemia and the risk of hypertension, renal diseases, cardiovascular disease, type 2 diabetes mellitus, insulin resistance, and obesity (16–19), it is not yet fully understood whether the SUA concentration might be a coexisting marker of these pathological processes or whether it has a causative role in the development of metabolic disturbances (20).

Serum uric acid levels in children and adolescents exhibit age-related variations, characterized by a notable rise after the age of 9-10 in both males and females. This observed trend could potentially be attributed to an increase in muscle mass, changes in nutritional metabolism, and the onset of puberty (21). Furthermore, recent studies have reported an increased incidence of healthcare problems associated with hyperuricemia, rising from 2-3 in 100,000 persons aged 0-9 years to 9-20 in 100,000 persons aged 10-19 years (22). Numerous studies have shown that high SUA levels are associated with MetS and its components even in children and adolescents (23–26). Because the reference SUA values for children and adolescents vary among countries and are generally lower than those for adults (27), large-scale studies describing sex- and age-specific variations in SUA levels among healthy children and adolescents are needed. A recent population-based study in Korea has shown an association between the prevalence of MetS and high levels of SUA in adolescence, in which high levels of SUA associated with overweight and obesity increased the risk of developing MetS. However, due to the limited study period and size, the study only provided ORs based on SUA quartiles, not ORs and changes in cardiometabolic risk values according to different SUA thresholds (28). Furthermore, high uric acid levels have been shown to correlate with inflammatory markers of MetS in obese or overweight individuals as well as in potentially healthy adults (29). Therefore, it is important to consider the impact of high uric acid levels on nonobese adolescent subjects as well.

In this study, we aimed to establish age- and sex-specific SUA reference values between 10 and 18 years of age with an expanded observation period of the KNHANES (2016–2018). Based on the age- and sex-specific SUA reference values, we attempted to determine the association between SUA levels and MetS risk after adjustment for age, sex and obesity. For the optimal diagnosis of hyperuricemia, we also used differential models to define hyperuricemia, for instance, age- and sex-specific percentile values or fixed or percentile values based on age.

## Materials and methods

### Subjects

This study was performed by using data from the KNHANES during 2016-2018. The KNHANES is a cross-sectional, nationally representative survey that is conducted annually by the Division of Chronic Disease Surveillance, Korean Centers for Disease Control and Prevention (KCDC). The subjects of the KNHANES are selected by stratified and multistage probability sampling of household units. The data consist of a health questionnaire, anthropometric measures, biochemical and clinical profiles, and a nutritional assessment. The KCDC publishes KNHANES reports annually, and the database is publicly accessible through the KNHANES website (http://knhanes.cdc.go.kr). All subjects provided informed consent at the time of data collection and the methods used in the KNHANES are performed in accordance with relevant guidelines and regulations. Details of the KNHANES were reported in a previous article (30). This study was approved by the Institutional Review Board of Hallym University Chuncheon Sacred Heart Hospital (IRB No. CHUNCHEON 2021-10-004).

**Figure 1:**
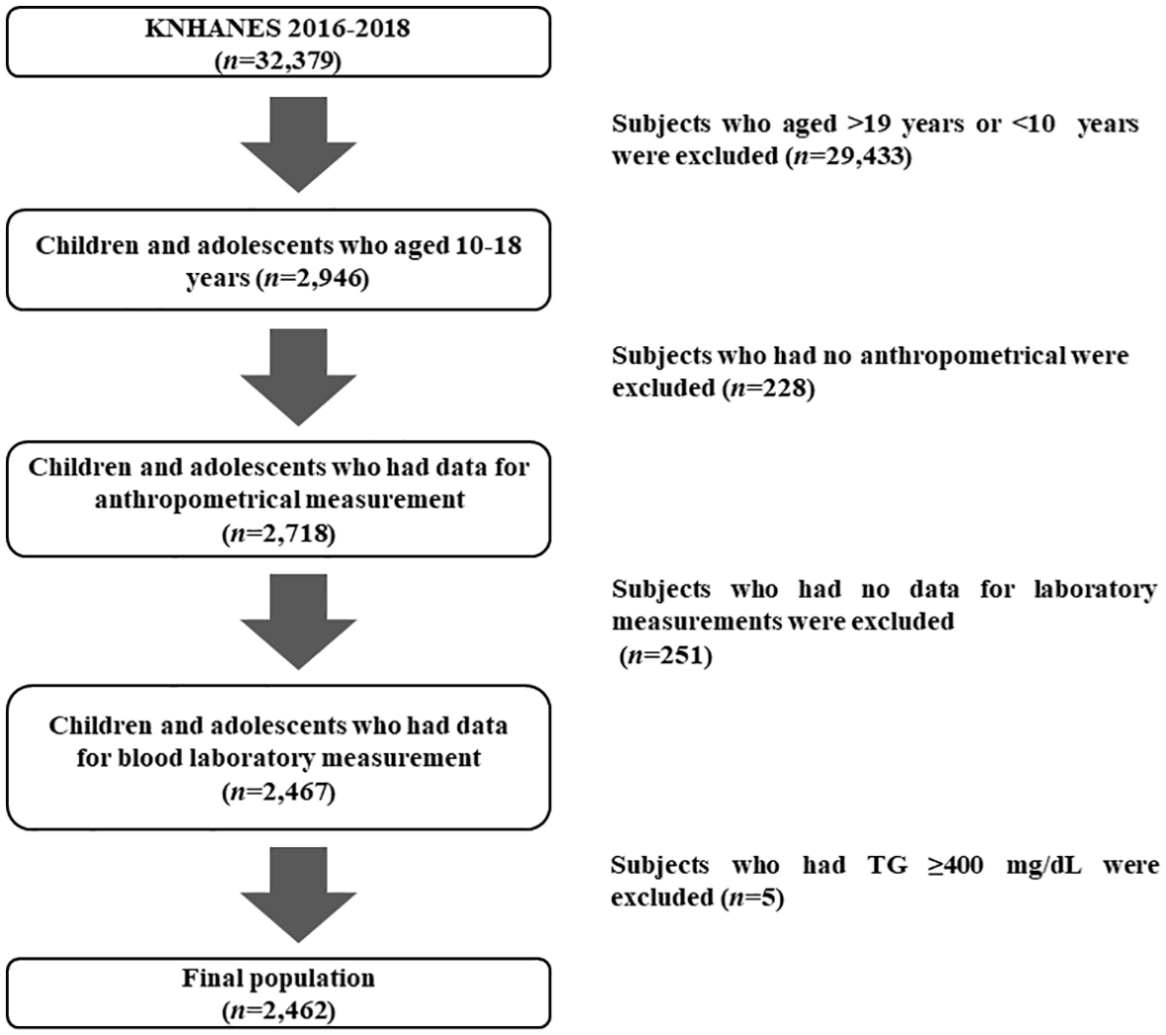
Flow chart of the study population (*n*=2,462).

The data in this study were from the KNHANES conducted from 2016 to 2018. Initially, subjects aged >19 years or <10 years were excluded (n=29,433) from the total sample of 32,379, and those with missing anthropometric data (n=228) and missing blood laboratory results were also excluded (n=251). Subjects with abnormal TG levels (≥400mg/dL) (n=5) were excluded based on the calculation of low-density lipoprotein cholesterol (LDL-C) by Friedewald’s equation. Thus, 2,462 subjects (1,300 males and 1,162 females) were included in the final analysis (**Figure 1**).

### Measurements

Anthropometric data and BP were measured by trained experts according to standardized protocols. Details of the anthropometric measurements have been described previously (31). SDSs were used for height, weight, BMI, and WC, which were calculated with the LMS methods using the 2017 Korean reference values (32). Normal weight, overweight, and obesity were defined as < 85th percentile, ≥ 85th percentile but < 95th percentile, and BMI ≥ 95th percentile, respectively, based on reference data from the Korean pediatric population (32). Venous blood samples were collected after the participants fasted for at least 8 h. Samples were immediately centrifuged, transported to a central laboratory (NeoDin Medical Institute, Seoul, Korea) and analyzed within 24 hours. Serum TC, HDL-c, TG, and glucose levels were measured enzymatically using a Hitachi 7600 automatic analyzer (Hitachi, Tokyo, Japan). LDL-c level were calculated with Friedewald’s equation (33).

### Collection of data on lifestyle parameters and socioeconomic status

Data on lifestyle-related parameters which consisted of smoking status, alcohol consumption, and physical activity level, and socioeconomic information were collected by questionnaires. Smoking was defined as having smoked more than five packs of cigarettes throughout one’s lifetime. Alcohol consumption was defined as the consumption of at least two alcoholic beverages/month during the previous year. Physical activity levels were determined based on the following criteria: Subjects were classified as exercising if they met any of the following conditions (1): engaging in intense physical activity for a duration of at least 30 minutes on at least three days per week (2), participating in moderate physical activity for a duration of at least 30 minutes on at least five days per week, or (3) engaging in walking for a duration of at least 30 minutes on at least five days per week. For socioeconomic information, household income was categorized as being within the lowest quartile or not. The residence area was divided into urban and rural areas.

### Definitions of MetS and its components

The definition of MetS followed the modified criteria of the NCEP-ATP III, as previously described in our published paper (34); subjects who met 3 of the following 5 criteria were defined as having MetS (1): a WC ≥90th percentile for age and sex according to the 2017 Korean growth chart (32) (2); an elevated BP, namely, an SBP or a DBP ≥90th percentile according to reference data from the Korean pediatric population (32) or treatment with antihypertensive medication (3); a fasting blood glucose level ≥100 mg/dL or treatment for type 2 diabetes mellitus (T2DM) (4); an elevated TG level (≥110 mg/dL); and (5) a low HDL-c level (<40 mg/dL). T2DM was diagnosed if one or more of the following criteria were met (1): self-reporting of the disease using a questionnaire (2), current medication or insulin use to manage T2DM, or (3) a fasting glucose level ≥ 126 mg/dL during the survey period.

### Statistical analysis

The basic characteristics consisted of continuous variables and categorical variables, with the mean ± standard deviation (SD) and frequencies or percentages (%) presented, respectively. Student’s t test was used to compare the means values of the demographic and biochemical characteristics. The chi-square (χ2) test was used to compare clinical categorical variables between boys and girls.

We obtained SUA percentile curves as a function of age as a continuous variable, stratified by sex using the LMS model to fit smoothed L (skew), M (median), and S (coefficient of variation) curves using the General Additive Model for Location Scale and Shape (GAMLSS) package version 4.2.6. of the R statistical package. The Box–Cox Cole and Green, gamma or inverse Gaussian distributions were fitted to the observed distribution of uric acid levels. Percentile curves for boys and girls were generated for the 3rd, 5th, 10th, 15th, 25th, 50th, 75th, 85th, 90th, 95th, and 97th percentiles. To analyze the association between SUA levels and MetS, we assessed Pearson’s correlation coefficients between SUA levels and other cardiometabolic risk factors with no adjustment in Model 1, adjustment for sex and age in Model 2, adjustment for sex, age, and obesity in Model 3, and adjustement for for sex, age, obesity, alcohol consumption, and creatinine in Model 4.

The adjusted mean values of the cardiometabolic risk factors were compared among the three groups using ANCOVA followed by Bonferroni’s *post-hoc* test after adjustment for sex, age, obesity, serum creatinine, alcohol consumption, smoking, physical activity, rural residence, household income, and diagnosis of hypertension, type 2 diabetes mellitus (T2DM), and dyslipidemia according to groups of SUA levels. In Model 1, the groups were classed by the percentile value according to sex and age differences (normal < 75th percentile; moderate-high, ≥ 75th and < 95th percentile; and high, ≥ 95th percentile). In Model 2, the groups were classified by the percentile value according to sex- and age-specific differences at 10-14 years of age in males and 10-12 years of age in females, at which SUA levels increased with age; after these ages, the fixed values according to the sex difference were used. In Model 3, the groups were classified based on a fixed value according to the sex difference.

We estimated the adjusted ORs of MetS and its components using 95% confidence intervals (CIs) among the normal, borderline high, and high SUA level groups by multiple logistic regression analysis. The classification criteria for SUA levels were the same as those for the ANCOVA and are presented in **Table 1**. The ORs of MetS and its components were determined using multiple logistic regression analysis after adjustment for sex, age, obesity, serum creatinine, alcohol consumption, smoking, physical activity, rural residence, household income, and diagnosis of hypertension, type 2 diabetes mellitus (T2DM), and dyslipidemia according to groups of SUA levels. P < 0.05 was considered statistically significant. All statistical analyses in this study were performed using the R statistical package version 3.5.1 (The R Foundation for Statistical Computing, Vienna, Austria).

**Table 1:** Adjusted means of cardiometabolic risk factors according to SUA level groups in Korean youth aged 10-18 years (*n*=2,462).

Model 1*						
Uric acid (mg/dL)						
	Boys			Girls		
	<75th percentile	≥75th percentile and <95th percentile	>95th percentile	<75th percentile	≥75th percentile and <95th percentile	>95th percentile
WC SDS	-0.21 ± 0.03	0.05 ± 0.05^a^	0.14 ± 0.09^b^	-0.24 ± 0.03	-0.06 ± 0.05^a^	0.38 ± 0.10^b,c^
SBP (mmHg)	109.88 ± 0.30	111.34 ± 0.54	114.20 ± 0.95^b,c^	105.20 ± 0.31	106.50 ± 0.58	105.13 ± 1.10
DBP (mmHg)	66.67 ± 0.29	65.37 ± 0.52	67.29 ± 0.92	65.80 ± 0.27	66.69 ± 0.51	66.34 ± 0.97
Glucose (mg/dL)	93.19 ± 0.24	92.39 ± 0.42	93.63 ± 0.74	91.11 ± 0.25	90.70 ± 0.47	91.18 ± 0.88
TC (mg/dL)	161.12 ± 0.90	163.37 ± 1.59	162.04 ± 2.83	168.30 ± 0.95	167.52 ± 1.80	173.59 ± 3.40
TG (mg/dL)	83.26 ± 1.54	85.06 ± 2.73	90.64 ± 4.85	87.45 ± 1.58	85.51 ± 2.98	105.08 ± 5.64^b,c^
HDL-c (mg/dL)	51.36 ± 0.32	49.62 ± 0.57^a^	49.55 ± 1.01	53.94 ± 0.34	52.78 ± 0.64	52.59 ± 1.22
LDL-c (mg/dL)	93.11 ± 0.77	96.74 ± 1.36	94.36 ± 2.42	96.87 ± 0.84	97.64 ± 1.59	99.98 ± 3.00
Model 2**						
	Group 1	Group 2	Group 3	Group 1	Group 2	Group 3
WC SDS	-0.22 ± 0.03	0.06 ± 0.06^a^	0.15 ± 0.09^b^	-0.24 ± 0.03	-0.06 ± 0.06^a^	0.36 ± 0.10^b,c^
SBP (mmHg)	109.88 ± 0.30	111.56 ± 0.53^a^	113.80 ± 1.01^b^	105.25 ± 0.30	106.40 ± 0.61	105.14 ± 1.10
DBP (mmHg)	66.66 ± 0.29	65.43 ± 0.51	67.48 ± 0.97	65.78 ± 0.27	66.98 ± 0.53	66.01 ± 0.96
Glucose (mg/dL)	93.17 ± 0.24	92.49 ± 0.41	93.61 ± 0.79	91.06 ± 0.24	90.90 ± 0.49	91.13 ± 0.88
TC (mg/dL)	160.89 ± 0.89	164.51 ± 1.57	160.44 ± 2.99	168.12 ± 0.94	168.09 ± 1.88	173.74 ± 3.40
TG (mg/dL)	83.29 ± 1.54	85.99 ± 2.70	87.89 ± 5.14	87.14 ± 1.56	86.46 ± 3.12	105.15 ± 5.62^b,c^
HDL-c(mg/dL)	51.32 ± 0.32	49.84 ± 0.56	49.26 ± 1.06	53.93 ± 0.34	52.78 ± 0.68	52.42 ± 1.22
LDL-c (mg/dL)	92.92 ± 0.76	97.48 ± 1.34	93.60 ± 2.56	96.77 ± 0.83	98.02 ± 1.66	100.30 ± 2.99
Model 3***						
	<6.7 mg/dL	≥6.7 and <8.1 mg/dL	≥8.1 mg/dL	<5.2 mg/dL	≥5.2 and <6.2 mg/dL	≥6.2 mg/dL
WC SDS	-0.19 ± 0.03	-0.01 ± 0.05^a^	0.22 ± 0.10^b^	-0.25 ± 0.03	-0.02 ± 0.05^a^	0.35 ± 0.11^b,c^
SBP (mmHg)	110.01 ± 0.30	111.12 ± 0.56	115.26 ± 1.17^b,c^	105.21 ± 0.31	106.33 ± 0.56	105.27 ± 1.20
DBP (mmHg)	66.55 ± 0.29	65.75 ± 0.54	67.60 ± 1.10	65.89 ± 0.27	66.43 ± 0.49	66.07 ± 1.05
Glucose (mg/dL)	93.13 ± 0.24	92.75 ± 0.44	93.08 ± 0.89	91.15 ± 0.25	90.57 ± 0.45	91.33 ± 0.96
TC (mg/dL)	161.85 ± 0.89	159.37 ± 1.66	168.78 ± 3.37^c^	168.44 ± 0.96	167.52 ± 1.74	172.69 ± 3.70
TG (mg/dL)	83.56 ± 1.53	83.41 ± 2.85	96.37 ± 5.79	86.95 ± 1.58	87.19 ± 2.88	108.47 ± 6.13^b,c^
HDL-c (mg/dL)	51.32 ± 0.32	49.29 ± 0.59^a^	50.59 ± 1.20	54.05 ± 0.34	52.65 ± 0.62	51.70 ± 1.33
LDL-c (mg/dL)	93.82 ± 0.76	93.40 ± 1.42	98.91 ± 2.89	97.00 ± 0.84	97.43 ± 1.53	99.29 ± 3.26

Data are presented as mean ± standard error (SE).

WC, waist circumference; SDS, standard deviation score; BMI, body mass index; SBP, systolic blood pressure; DBP, diastolic blood pressure; TC, total cholesterol; TG, triglyceride; HDL-c, high-density lipoprotein cholesterol; LDL-c, low-density lipoprotein cholesterol.

In Models 1 and 2, adjusted mean ± SE was determined using analysis of covariance for MetS and its components after adjustment for sex, age, obesity, alcohol consumption, smoking, physical activity, rural residence, household income, and diagnosis of hypertension, type 2 diabetes mellitus (T2DM), and dyslipidemia according to groups of SUA levels.

In Model 3, adjusted mean ± SE was determined using analysis of covariance for MetS, and its components were determined using multiple logistic regression analysis after adjustment for sex, age, obesity, serum creatinine, alcohol consumption, smoking, physical activity, rural residence, household income, and diagnosis of hypertension, type 2 diabetes mellitus (T2DM), and dyslipidemia according to groups of SUA levels.

*: In Model 1, the groups are classed according to the percentile calculated using cubic spline methods.

**: In Model 2, group 1 was classified according to SUA levels: i) <5.0 mg/dL in boys aged 10-11 years, ii) <5.6 mg/dL in boys aged 11-12 years, iii) <6.1 mg/dL in boys aged 12-13 years, iv) <6.6 mg/dL in boys aged 13-14 years, and v) <7.1 mg/dL in boys aged 14-18 years.

**: In Model 2, group 1 was classified according to SUA levels: i) <4.9 mg/dL in girls aged 10-11 years, <5.1 mg/dL in girls aged 11-12 years, and <5.3 mg/dL in girls aged 12-18 years.

**: In Model 2, group 2 was classified according to SUA levels: i) 5.0 ≥ and <6.0 mg/dL in boys aged 10-11 years, ii) ≥5.6 and <6.7 mg/dL in boys 11-12 aged years, iii) ≥6.1 and <7.4 mg/dL in boys aged 12-13 years, iv) ≥6.6 and <8.0 mg/dL in boys aged 13-14 years, and v) ≥7.1 and <8.3 mg/dL in boys aged 14-18 years.

**: In Model 2, group 2 was classified according to SUA levels: i) ≥4.9 and <6.0 mg/dL in girls aged 10-11 years, ii) ≥5.1 and <6.7 mg/dL in girls 11-12 aged years, and iii) ≥5.3 and <6.2 mg/dL in girls aged 12-18 years.

**: In Model 2, group 3 was classified according to SUA levels: i) ≥6.0 mg/dL in boys aged 10-11 years, ii) ≥6.7 mg/dL in boys aged 11-12 years, iii) ≥7.4 mg/dL in boys aged 12-13 years, iv) <8.0 mg/dL in boys aged 13-14 years, and v) ≥8.3 mg/dL in boys aged 14-18 years.

**: In Model 2, group 3 was classified according to SUA levels: i) ≥5.7 mg/dL in girls aged 10-11 years, ii) ≥6.0 mg/dL in girls aged 11-12 years, and iii) ≥6.2 mg/dL in girls aged 12-18 years. ***: In Model 3, each group was classified based on SUA levels, using the 75th and 95th percentiles as reference values for each gender, while disregarding age.

a : The difference was estimated between group 1 and group 2 using analysis of covariance with Bonferroni’s post-hoc test.

b : The difference was estimated between group 1 and group 3 using analysis of covariance with Bonferroni’s post-hoc test.

c : The difference was estimated between group 2 and group 3 using analysis of covariance with Bonferroni’s post-hoc test.

## Results

### Clinical characteristics of the study participants

The characteristics of the study participants are summarized in **Table 2**. The mean ages of the subjects were 14.42 ± 2.58 and 14.49 ± 2.57 years for males and females, respectively. The weight, waist circumferences (WC) and body mass index (BMI) standard deviation scores (SDSs) did not differ between males and females. However, the systolic blood pressure (SBP), serum glucose, total cholesterol (TC), triglyceride (TG), SUA, BUN, and creatinine levels differed between boys and girls. In the health questionnaire survey, there were sex differences in alcohol consumption, smoking status, and physical activity levels.

**Table 2:** Clinical characteristics of study population (*n*=2,462).

	Boys	Girls	*P*
	*n*=1,300	*n*=1,162	
Age (years)	14.42 ± 2.58	14.49 ± 2.57	0.499
Height SDS	0.32 ± 1.07	0.25 ± 1.03	0.150
Weight SDS	0.20 ± 1.26	0.11 ± 1.20	0.087
WC SDS	-0.13 ± 1.18	-0.17 ± 1.13	0.404
BMI SDS	0.05 ± 1.34	-0.02 ± 1.29	0.219
SBP (mmHg)	110.53 ± 10.02	105.46 ± 9.11	<0.001
DBP (mmHg)	66.43 ± 9.48	66.02 ± 8.12	0.244
Glucose (mg/dL)	93.05 ± 8.57	91.03 ± 7.56	<0.001
TC (mg/dL)	161.68 ± 27.42	168.46 ± 27.50	<0.001
TG (mg/dL)	50.84 ± 10.17	53.62 ± 10.21	<0.001
HDL-c (mg/dL)	84.21 ± 48.14	88.09 ± 46.79	0.043
LDL-c (mg/dL)	94.00 ± 23.59	97.21 ± 24.32	0.001
Uric acid (mg/dL)	5.89 ± 1.31	4.62 ± 0.90	<0.001
BUN (mg/dL)	12.99 ± 3.07	11.49 ± 2.72	<0.001
Creatinine (mg/dL)	0.74 ± 0.17	0.61 ± 0.10	<0.001
Alcohol consumption (%)	316 (24.3%)	233 (20.1%)	0.013
Smoking	134 (10.3%)	50 (4.3%)	<0.001
Physical activity	345 (26.5%)	220 (18.9%)	<0.001
Rural residence	171 (13.2%)	169 (14.5%)	0.347
Household income ≤ 1st quartile	127 (9.8%)	124 (10.7%)	0.502
Diagnosis of Hypertension	0 (0%)	0 (0%)	>0.999
Diagnosis of T2DM	1 (0.1%)	0 (0%)	>0.999
Diagnosis of dyslipidemia	0 (0%)	0 (0%)	>0.999

Data are presented as mean ± standard deviation (SD). WC, waist circumference; SDS, standard deviation score; BMI, body mass index; SBP, systolic blood pressure; DBP, diastolic blood pressure; TC, total cholesterol; TG, triglycerides; HDL-c, high-density lipoprotein cholesterol; LDL-c, low-density lipoprotein cholesterol; BUN, blood urea nitrogen; T2DM, type 2 diabetes mellitus.

### Sex- and age-specific SUA percentile values in Korean youth aged 10-18 years

**Figure 2:**
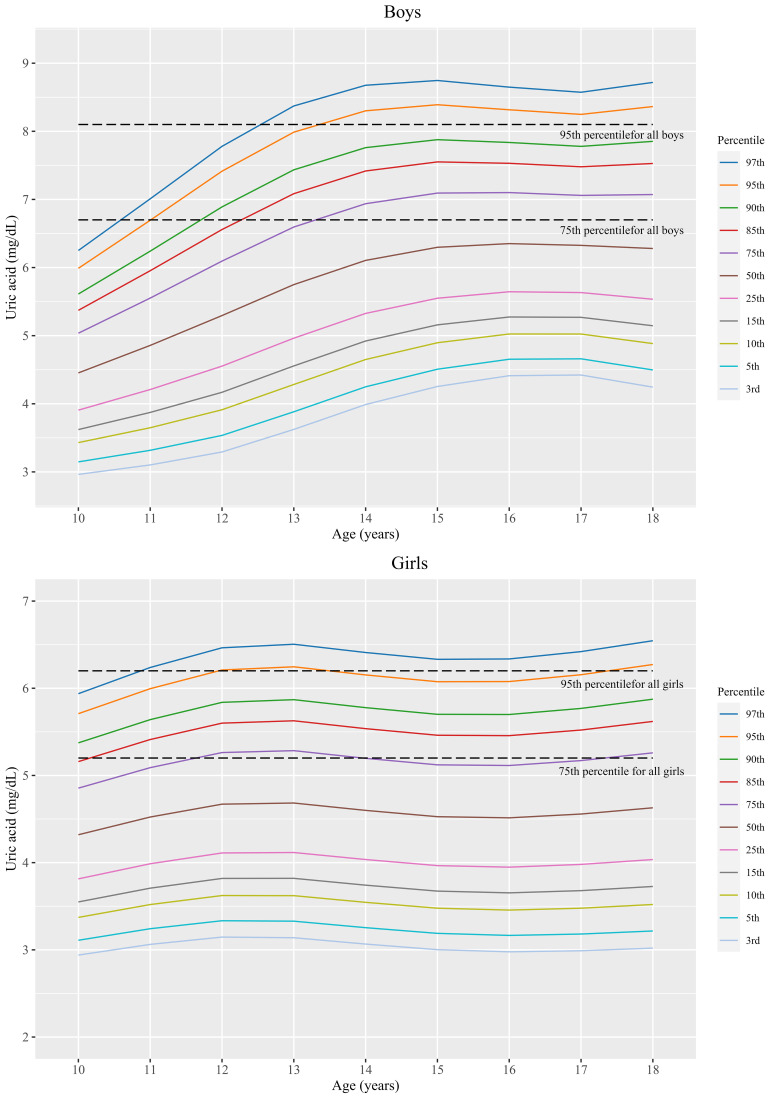
Sex- and age-specific SUA percentiles in Korean youth aged 10 to 18 years (*n*=2,462).

The sex- and age-specific SUA distributions are shown in **Figure 2**. The percentile values for each age and sex category, corresponding to the LMS variables, are summarized in **Table 3**. SUA values varied considerably according to sex and age. The overall SUA levels were higher in boys than girls; this tendency was generally similar across percentile groups. The increase in SUA levels according to age was more prominently observed in the males (from 10 to 14 years old) than in females (from 10 to 12 years old). The overall SUA levels were relatively constant over 14 years of age in males and 12 years of age in females.

**Table 3:** Sex- and age-specific percentile values of SUA in Korean youth aged 10-18 years (*n*=2,462).

Boys															
Age	*n*	L	M	S	3rd	5th	10th	15th	25th	50th	75th	85th	90th	95th	97th
10^1^	143	0.494	4.453	0.184	3.0	3.1	3.4	3.6	3.9	4.5	5.0	5.4	5.6	6.0	6.3
11^1^	157	0.494	4.857	0.201	3.1	3.3	3.6	3.9	4.2	4.9	5.6	6.0	6.2	6.7	7.0
12^1^	141	0.494	5.295	0.212	3.3	3.5	3.9	4.2	4.6	5.3	6.1	6.6	6.9	7.4	7.8
13^1^	174	0.494	5.750	0.206	3.6	3.9	4.3	4.6	5.0	5.7	6.6	7.1	7.4	8.0	8.4
141	138	0.494	6.105	0.192	4.0	4.2	4.6	4.9	5.3	6.1	6.9	7.4	7.8	8.3	8.7
15^1^	131	0.494	6.297	0.178	4.3	4.5	4.9	5.2	5.5	6.3	7.1	7.6	7.9	8.4	8.7
16^1^	123	0.494	6.351	0.167	4.4	4.7	5.0	5.3	5.6	6.4	7.1	7.5	7.8	8.3	8.6
17^1^	157	0.494	6.325	0.164	4.4	4.7	5.0	5.3	5.6	6.3	7.1	7.5	7.8	8.2	8.6
18^1^	136	0.494	6.279	0.178	4.2	4.5	4.9	5.1	5.5	6.3	7.1	7.5	7.9	8.4	8.7
All^2^	1,300				3.6	3.8	4.2	4.5	5.0	5.9	6.7	7.3	7.6	8.1	8.6
Girls															
10^1^	130	0.544	4.320	0.176	2.9	3.1	3.4	3.5	3.8	4.3	4.9	5.2	5.4	5.7	5.9
11^1^	123	0.544	4.523	0.178	3.1	3.2	3.5	3.7	4.0	4.5	5.1	5.4	5.6	6.0	6.2
12^1^	133	0.544	4.671	0.180	3.1	3.3	3.6	3.8	4.1	4.7	5.3	5.6	5.8	6.2	6.5
13^1^	136	0.544	4.684	0.182	3.1	3.3	3.6	3.8	4.1	4.7	5.3	5.6	5.9	6.2	6.5
141	127	0.544	4.599	0.184	3.1	3.3	3.5	3.7	4.0	4.6	5.2	5.5	5.8	6.2	6.4
15^1^	133	0.544	4.527	0.186	3.0	3.2	3.5	3.7	4.0	4.5	5.1	5.5	5.7	6.1	6.3
16^1^	120	0.544	4.514	0.189	3.0	3.2	3.5	3.7	3.9	4.5	5.1	5.5	5.7	6.1	6.3
17^1^	136	0.544	4.558	0.191	3.0	3.2	3.5	3.7	4.0	4.6	5.2	5.5	5.8	6.2	6.4
18^1^	124	0.544	4.629	0.193	3.0	3.2	3.5	3.7	4.0	4.6	5.3	5.6	5.9	6.3	6.5
All^2^	1,162				3.0	3.2	3.5	3.8	4.0	4.6	5.2	5.6	5.8	6.2	6.4

^1^: The respective percentiles were determined using penalized likelihood in nonlinear regression to fit the curves as cubic splines.

^2^: The respective percentiles were determined using conventional method.

### Differences in SUA levels according to BMI

**Figure 3:**
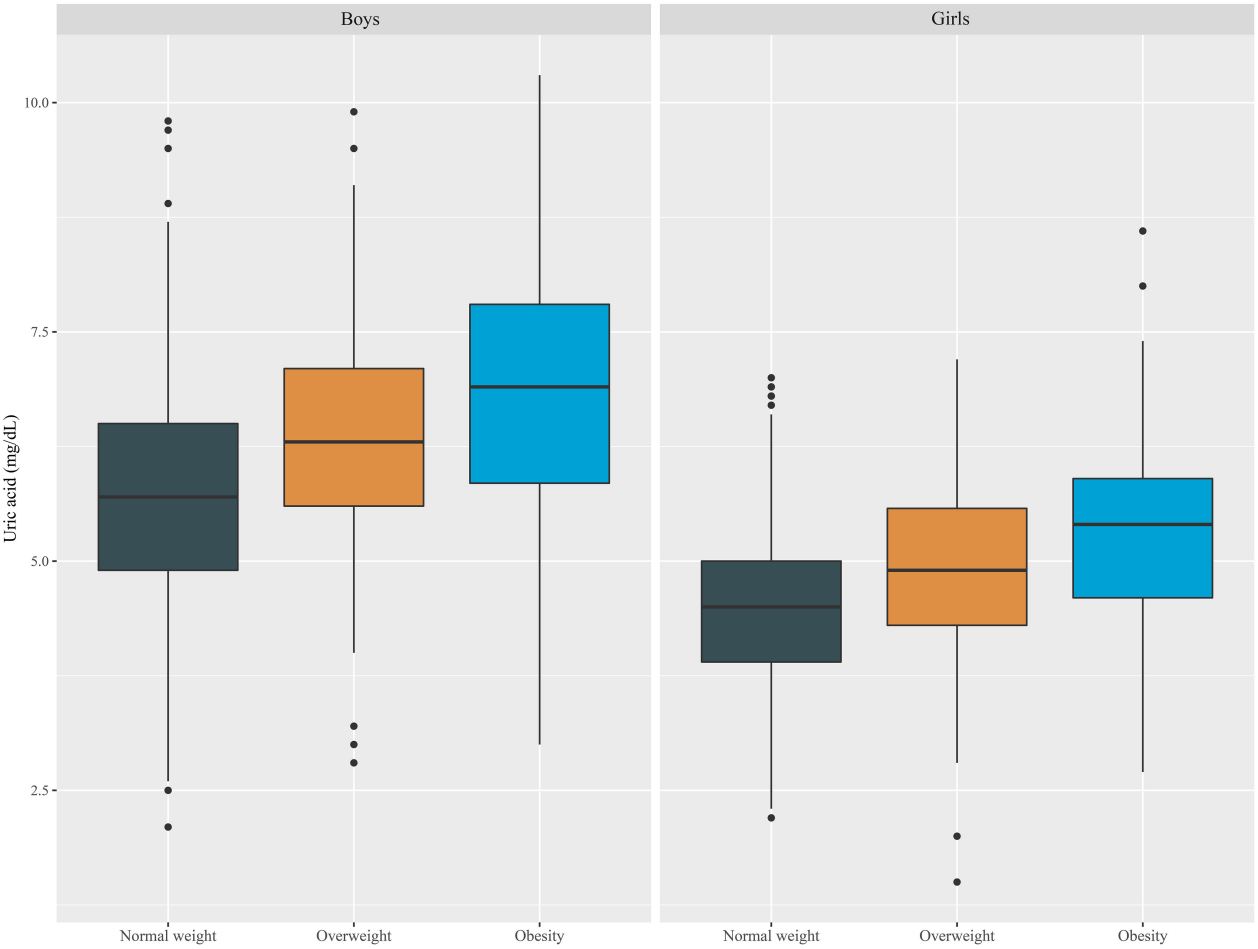
Boxplot of SUA levels according to sex and obesity status in Korean youth aged 10 to 18 years (*n*=2,462).

The degree of obesity was defined as follows based on Korean reference data for age and sex as described in materials and methods. Differences in SUA levels were not only attributable to sex but also tended to increase in proportion to obesity in both males and females (**Figure 3**).

### Unadjusted and adjusted correlation analysis of the relationship between SUA levels and cardiometabolic risk factors

The correlations between the SUA levels and cardiometabolic risk factors are shown in **Table 4**. An elevated SUA level was positively correlated with various cardiometabolic risk factors, such as the WC SDS, SBP, diastolic blood pressure (DBP), TC, TG, and HDL-c levels, in unadjusted Model 1, most of which, except the TC level in model 2 and the TC level and DBP in model 3 and model 4, remained significant even after adjusting for sex and age in model 2, sex, age and obesity in model 3 and sex, age, obesity, alcohol consumption, and creatinine in model 4. A statistically significant inverse correlation was found between SUA levels and HDL-c levels in All models.

**Table 4:** Unadjusted and adjusted correlations between SUA levels and cardiometabolic risk factors in Korean youth aged 10-18 years (*n*=2,462).

	Uric acid (mg/dL)							
	Model 1		Model 2		Model 3		Model 4	
	r	*P*	r	*P*	r	*P*	r	*P*
WC SDS	0.300	<0.001	0.296	<0.001	0.123	<0.001	0.163	<0.001
SBP (mmHg)	0.313	<0.001	0.182	<0.001	0.114	<0.001	0.099	<0.001
DBP (mmHg)	0.136	<0.001	0.067	<0.001	0.026	0.206	0.003	0.894
Glucose (mg/dL)	0.024	0.233	0.011	0.577	-0.024	0.230	-0.026	0.199
TC (mg/dL)	-0.058	0.004	0.013	0.517	-0.030	0.131	-0.022	0.280
TG (mg/dL)	0.093	<0.001	0.130	<0.001	0.065	0.001	0.070	<0.001
HDL-c (mg/dL)	-0.249	<0.001	-0.197	<0.001	-0.130	<0.001	-0.128	<0.001
LDL-c (mg/dL)	0.003	0.888	0.047	0.020	-0.006	0.756	-0.001	0.978

WC, waist circumference; SDS, standard deviation score; BMI, body mass index; SBP, systolic blood pressure; DBP, diastolic blood pressure; TC, total cholesterol; TG, triglycerides; HDL-c, high-density lipoprotein cholesterol; LDL-c, low-density lipoprotein cholesterol.

Model 1: The statistical significance was determined using Pearson correlation analysis without adjustment.

Model 2: The statistical significance was determined using Pearson correlation analysis after adjustment for sex and age.

Model 3: The statistical significance was determined using Pearson correlation analysis after adjustment for sex, age, and obesity.

Model 4: The statistical significance was determined using Pearson correlation analysis after adjustment for sex, age, obesity, alcohol consumption, and creatinine.

### Adjusted means values of cardiometabolic risk factors according to SUA levels groups

The adjusted mean values of the cardiometabolic risk factors according to SUA level groups are shown in **Table 1**. The mean values of the cardiometabolic risk factors were estimated using analysis of covariance (ANCOVA) followed by Bonferroni’s *post hoc* test. Details of the Models for classifications are presented in **Table 1**. All three models showed some increase in metabolic risk due to elevated SUA levels. Among the various risk factors, the WC SDS increased with an increased SUA level, which was similarly observed in both boys and girls. However, the increased SBP, low HDL-c, or high TC due to SUA level was observed in boys, but the increased TG according to the SUA level was observed in girls.

### Adjusted odds ratio of MetS and its components according to SUA level groups

To evaluate the OR of MetS and its components according to SUA levels, we used the same classification criteria that were applied in the analysis of the adjusted mean values of the cardiometabolic risk factors. The adjusted OR of MetS was significantly increased in the high SUA groups in Model 1, Model 2, and Model 3. In particular, even the moderately-high uric acid level group had significantly higher ORs for low HDL-c and MetS than the normal references values in Model 2 and 3. Adjusted ORs for elevated WC and TG levels were increased and low HDL-c for the high uric acid level group in all three models. Moreover, the adjusted OR for MetS was considerably increased in the moderately high as well as high SUA level groups in models 2 and 3.

The results from a sex-specific analysis revealed significant differences in the impact of MetS risk factors between boys and girls. In boys, ORs for reduced HDL-c were markedly increased across all models, whereas in girls, significant increases in OR were observed for elevated WC and TG levels, as well as reduced HDL-c, in correlation with SUA levels. The increase in OR for MetS associated with SUA was distinctly higher in girls but did not show a significant rise in boys, which is summarized in **Table 5**.

**Table 5:** Adjusted odds ratio of metabolic syndrome (MetS) and its components according to groups of SUA levels in Korean youth aged 10-18 years (*n*=2,462).

Model 1*									
SUA	All participants			Boys			Girls		
	<75th percentile	≥75th percentile and <95th percentile	≥95th percentile	<75th percentile	≥75th percentile and <95th percentile	≥95th percentile	<75th percentile	≥75th percentile and <95th percentile	≥95th percentile
Elevated WC	Reference	1.04 (0.64-1.68)	2.16 (1.17-4.00)	Reference	0.91 (0.44-1.86)	1.73 (0.73-4.13)	Reference	1.24 (0.64-2.43)	3.34 (1.37-8.14)
Elevated BP	Reference	1.03 (0.83-1.28)	1.11 (0.78-1.57)	Reference	0.91 (0.68-1.22)	1.17 (0.74-1.86)	Reference	1.18 (0.86-1.62)	1.02 (0.59-1.76)
Elevated glucose	Reference	1.11 (0.47-2.60)	1.31 (0.41-4.25)	Reference	0.69 (0.19-2.51)	1.55 (0.38-6.28)	Reference	1.43 (0.40-5.07)	1.06 (0.12-9.66)
Elevated TG	Reference	1.13 (0.89-1.43)	1.55 (1.08-2.22)	Reference	1.32 (0.96-1.82)	1.25 (0.98-1.11)	Reference	0.95 (0.67-1.35)	2.13 (1.24-3.64)
Reduced HDL-c	Reference	1.58 (1.15-2.17)	1.69 (1.06-2.69)	Reference	1.56 (1.05-2.39)	1.31 (0.72-2.39)	Reference	1.63 (0.93-2.85)	2.89 (1.38-6.05)
MetS	Reference	1.53 (0.98-2.40)	2.15 (1.25-3.71)	Reference	1.53 (0.87-2.68)	1.15(0.55-2.39)	Reference	1.54 (0.68-3.48)	6.39 (2.67-15.27)
Model 2**									
SUA	Group 1	Group 2	Group 3	Group 1	Group 2	Group 3	Group 1	Group 2	Group 3
Elevated WC	Reference	1.12 (0.69-1.82)	2.08 (1.12-3.85)	Reference	1.11 (0.54-2.25)	1.73 (0.71-4.19)	Reference	1.19 (0.60-2.37)	3.12 (1.31-7.43)
Elevated BP	Reference	1.10 (0.89-1.36)	1.02 (0.71-1.47)	Reference	0.97 (0.73-1.30)	1.10 (0.68-1.80)	Reference	1.28 (0.92-1.77)	0.93 (0.53-1.61)
Elevated glucose	Reference	1.15 (0.49-2.71)	1.42 (0.44-4.62)	Reference	0.66 (0.18-2.42)	1.75 (0.43-7.20)	Reference	1.66 (0.48-5.71)	1.13 (0.13-10.24)
Elevated TG	Reference	1.20 (0.95-1.52)	1.50 (1.04-2.18)	Reference	1.37 (1.00-1.89)	1.09 (0.64-1.87)	Reference	1.00 (0.70-1.44)	2.24 (1.31-3.83)
Reduced HDL-c	Reference	1.54 (1.12-2.13)	1.63 (1.01-2.62)	Reference	1.52 (1.03-2.26)	1.26 (0.67-2.35)	Reference	1.57 (0.89-2.78)	2.76 (1.32-5.78)
MetS	Reference	1.69 (1.08-2.64)	2.08 (1.20-3.62)	Reference	1.61 (0.92-2.82)	1.04 (0.49-2.23)	Reference	1.84 (0.82-4.13)	6.63 (2.78-15.80)
Model 3***									
SUA	<6.7 mg/dL in boys <5.2 mg/dL in girls	≥6.7 and <8.1 mg/dL in boys ≥5.2 and <6.2 mg/dL in girls	≥8.1 mg/dL in boys ≥6.2 mg/dL in girls	<6.7 mg/dL in	≥6.7 and <8.1 mg/dL	≥8.1 mg/dL	<5.2 mg/dL	≥5.2 and <6.2 mg/dL	≥6.2 mg/dL
Elevated WC	Reference	1.50 (0.92-2.43)	4.05 (2.01-8.19)	Reference	1.63 (0.77-3.45)	4.35 (1.50-12.63)	Reference	1.28 (0.66-2.51)	4.24 (1.64-10.95)
Elevated BP	Reference	1.04 (0.84-1.29)	1.12 (0.76-1.66)	Reference	0.89 (0.66-1.20)	1.21 (0.71-2.06)	Reference	1.14 (0.84-1.56)	0.91 (0.50-1.67)
Elevated glucose	Reference	1.08 (0.45-2.60)	1.44 (0.38-5.48)	Reference	0.62 (0.16-2.38)	1.30 (0.25-6.82)	Reference	1.35 (0.38-4.77)	1.59 (0.17-14.72)
Elevated TG	Reference	1.18 (0.93-1.50)	2.07 (1.38-3.10)	Reference	1.15 (0.81-1.63)	1.70 (0.96-3.02)	Reference	1.11 (0.79-1.56)	2.50 (1.40-4.49)
Reduced HDL-c	Reference	1.68 (1.22-2.32)	2.00 (1.21-3.31)	Reference	1.56 (1.04-2.34)	1.26 (0.64-2.48)	Reference	1.66 (0.95-2.88)	3.87 (1.80-8.31)
MetS	Reference	1.70 (1.08-2.68)	3.25 (1.84-5.76)	Reference	1.60 (0.91-2.82)	1.71 (0.79-3.71)	Reference	1.50 (0.66-3.41)	8.48 (3.43-20.97)

SUA, serum uric acid; WC, waist circumference; SDS, standard deviation score; BMI, body mass index; SBP, systolic blood pressure; DBP, diastolic blood pressure; TC, total cholesterol; TG, triglycerides; T2DM, HDL-c, high-density lipoprotein cholesterol; MetS, metabolic syndrome.

In models 1 and 2, the odds ratio of MetS and its components was determined using multiple logistic regression analysis after adjustment for sex, age, obesity, alcohol consumption, smoking, physical activity, rural residence, household income, and diagnosis of hypertension, type 2 diabetes mellitus (T2DM), and dyslipidemia according to groups of SUA levels.

*: In Model 1, the groups are classed according to the sex- and age-specific percentile of SUA calculated using cubic spline methods.

**: In Model 2, group 1 was classified according to SUA levels: i) <5.0 mg/dL in boys aged 10-11 years, ii) <5.6 mg/dL in boys 11-12 aged years, iii) <6.1 mg/dL in boys aged 12-13 years, iv) <6.6 mg/dL in boys aged 13-14 years, and v) <7.1 mg/dL in boys aged 14-18 years.

**: In Model 2, group 1 was classified according to SUA levels: i) <4.9 mg/dL in girls aged 10-11 years, <5.1 mg/dL in girls aged 11-12 years, and <5.3 mg/dL in girls aged 12-18 years.

**: In Model 2, group 2 was classified according to SUA levels: i) 5.0 ≥ and <6.0 mg/dL in boys aged 10-11 years, ii) ≥5.6 and <6.7 mg/dL in boys 11-12 aged years, iii) 6.1 and <7.4 mg/dL in boys aged 12-13 years, iv) ≥6.6 and <8.0 mg/dL in boys aged 13-14 years, and v) ≥7.1 and <8.3 mg/dL in boys aged 14-18 years.

**: In Model 2, group 2 was classified according to SUA levels: i) ≥4.9 and <6.0 mg/dL in girls aged 10-11 years, ii) ≥5.1 and <6.7 mg/dL in girls 11-12 aged years, and iii) ≥5.3 and <6.2 mg/dL in girls aged 12-18 years.

**: In model 2, group 3 was classified according to SUA levels: i) ≥6.0 mg/dL in boys aged 10-11 years, ii) ≥6.7 mg/dL in boys 11-12 aged years, iii) ≥7.4 mg/dL in boys aged 12-13 years, iv) <8.0 mg/dL in boys aged 13-14 years, and v) ≥8.3 mg/dL in boys aged 14-18 years.

**: In model 2, group 3 was classified according to SUA levels: i) ≥5.7 mg/dL in girls aged 10-11 years, ii) ≥6.0 mg/dL in girls aged 11-12 years, and iii) ≥6.2 mg/dL in girls aged 12-18 years.

***: In model 3, groups were classified into 3 groups according to the sex-specific percentiles of SUA. The odds ratio of MetS and its components was determined using multiple logistic regression analysis after adjustment for sex, age, obesity, serum creatinine, alcohol consumption, smoking, physical activity, rural residence, household income, and diagnosis of hypertension, type 2 diabetes mellitus (T2DM), and dyslipidemia according to groups of SUA levels.

## Discussion

In this study, we established age- and sex-specific SUA reference curves for Korean adolescents between 10 and 18 years of age; there were differences in SUA levels between males and females according to age change. As the degree of obesity increased, increasing SUA levels were observed in both males and females. To determine the diagnostic upper limits for SUA levels associated with cardiometabolic risks in children and adolescents, we assessed the adjusted mean values and ORs for cardiometabolic risks using various models. The results indicated that the increase in cardiometabolic risk, including increased WC SDSs, SBP, and HDL-c levels, was similarly increased with moderate to high uric acid levels, as determined by the reference values established for all ages (Model 3) compared to the reference values based on age-specific percentile values (Model 1) or age-specific reference values (Model 2). These results suggest that age-related changes in uric acid levels may not be a major consideration in the assessment of MetS risk.

The reference curves of SUA level were generally higher in males than in females, and an age-dependent increase was more clearly observed in males from the ages of 10 to 14 years, while in females, there was a gradual increase from the ages of 10 to 12 years, which was consistent with previous studies (21, 35). This difference may be attributed to several factors, including the increase in muscle content and nutrition metabolism that occurs during growth. The change in sex hormones during puberty, specifically the increase in testosterone and estrogen, may also play a role in the difference in uric acid levels between males and females. During adolescence, an increase in testosterone levels promotes an increase in uric acid levels, possibly through the promotion of muscle anabolism, which is a major source of purine. This can lead to an increase in SUA (36, 37). Additionally, testosterone may inhibit the excretion of uric acid, while estrogen may promote it, which also affects SUA levels (36, 37).

The association between high SUA levels and MetS has been highlighted in several studies. One important mechanism has been suggested that hyperuricemia impairs insulin-mediated glucose uptake in skeletal muscle by reducing blood flow and the release of nitric oxide release from endothelial cells (38, 39). The other mechanism is involved in uric acid-induced inflammatory and oxidative changes in adipocytes, of which the development of hyperuricemia is influenced by oxidative stress and consequently results in insulin resistance via redox-dependent signaling (12, 40). Furthermore, hyperuricemia has been considered crucial in the process of adipogenesis and vice versa (41). Regarding hypertension, high SUA levels have been shown to lead to renal vasoconstriction mediated by a decrease in endothelial nitric oxide and the activation of the renin-angiotensin system (39, 42). Not only have experimental studies demonstrated the underlying mechanisms of uric acid in the development of MetS, but several epidemiological studies have also shown a strong correlation between SUA levels and the occurrence of MetS, in adolescents as well as adults (19, 23, 25, 43). Although sex- and race-specific differences in the relationship between uric acid levels and MetS have been reported in adolescents (44), it has been generally accepted that uric acid levels are positively associated with the occurrence of MetS in several nationwide populational studies, including studies from Taiwan (45), Japan (46), Italy (47), and the USA (23, 48). Similarly, recent Korean studies have also shown a positive correlation between SUA levels and cardiometabolic risk factors based on the KNHANES database (28, 49).

In MetS, hepatic involvement is commonly observed as nonalcoholic fatty liver disease (NAFLD) (6). Pathological hepatosteatosis with chronic inflammation leads to impaired glucose and lipid metabolism, accompanied by increased oxidative stress, endothelial dysfunction, and hypercoagulability, which can eventually result in cardiovascular disease (50). Hyperuricemia has been considered an important risk factor for NAFLD and has influenced the progression and histological severity by triggering endothelial dysfunction, insulin resistance, oxidative stress, and systemic inflammation (51). Moreover, in patients with metabolic syndrome, serum bcl-2 levels, which are associated with NAFLD activity, have been reported to be closely related to HOMA, BMI, and serum uric acid levels (52). Dietary interventions, such as limiting purine-rich foods, reducing sugar and alcohol intake, and increasing vegetable and water consumption, have been proposed to lower uric acid levels in MetS. Various natural remedies for uric acid control in adults are also being developed (53). However, there have been no reported attempts to reduce hyperuricemia associated with MetS during the growth period. Based on the findings of various studies on the role of uric acid and the associated risks of MetS in pediatric populations, it might be necessary to evaluate the effectiveness and safety of interventions aimed at reducing hyperuricemia in children and adolescents in future studies.

We analyzed various models of uric acid standard values such as sex- and age-specific percentiles, age-specific uric acid values, and sex-specific uric acid values. Incorporating obesity as a variable in the adjusted OR analysis and ANCOVA allowed for a more precise understanding of the relationship among SUA levels, obesity, and cardiometabolic risk factors. Although SUA is not a specific marker of obesity, recent studies have shown a relationship between SUA levels and measures of adiposity in obese children (26, 54). Similarly, our study also revealed that SUA levels tend to increase with obesity (**Figure 3**), but they are also associated with cardiometabolic risk factors regardless of obesity status; as shown in **Table 4**, cardiometabolic risk factors, including the WC SDS, SBP and TG and HDL-c levels, were closely associated with SUA levels after adjustment for sex, age, and obesity. Despite being conducted with adult data, multiple studies have demonstrated that elevated SUA levels are correlated with an increased risk of metabolic and cardiovascular diseases, even in nonobese individuals (55–57). Therefore, this highlights the importance of monitoring uric acid levels in individuals with or without obesity, as it may be a risk factor for other health issues.

The strengths of this study include that a more extensive database was utilized compared to previous studies, which enables a more comprehensive analysis of the relationship between SUA levels and cardiometabolic risk factors. As shown in **Tables 4** and **5**, high SUA levels were associated with changes in the WC SDS, SBP, TG, and HDL-c levels, which are related to MetS in overall subjects. These results were partially consistent with previous findings; however, we observed more compelling results on separating analysis by sex. The ANCOVA of cardiometabolic risk factors according to SUA levels showed that the mean WC SDS and SBP increased significantly in males, whereas the mean WC SDS and TG increased in females (**Table 1**). Moreover, the multivariate logistic regression analysis highlighted marked gender differences in the OR for MetS associated with hyperuricemia (**Table 5**). Concerning those sex differences, a recent study also noted that the increase in OR for MetS related to SUA levels was greater in females than in males. However, apart from TG, other cardiovascular risk factors showed no significant sex differences (28). The disparities in SUA levels between boys and girls during adolescence might be associated with sex hormonal effects as aforementioned mechanisms. However, we consistently observed a significant increase in the OR for MetS associated with elevated SUA levels in girls, but not in boys after adjustment for confounding variables including age, sex, obesity, as well as serum creatinine levels. An increase in ORs for low HDL-c and central obesity was only noted in males (**Table 5**). Several lines of studies in adult populations have also shown that the association between high SUA levels and the risk of MetS differs by sex. Notably, the impacts of SUA levels on fasting glucose levels, NAFLD, cardiovascular events, and health outcomes were higher in females than in males (58–61). Therefore, it is suggested that prognostic and risk evaluations should be considered differently between boys and girls in the clinical assessment for high SUA levels linked to MetS during adolescence.

Our study has some limitations, one of which was the limited ethnicities and periods included for analysis. Although we analyzed a larger sample than previous studies, it is still not sufficient to generalize the results, particularly to clarify ethnicity- or sex- differences in components of metabolic risk; therefore, further research based on a broader period and study sample is needed in future studies. Additionally, our study relied on self-reported measurements of physical activity and dietary intake, which may be subject to recall and measurement biases. We did not take into account other potential causes that may contribute to an increase in SUA levels into account, such as pubertal stage, dietary fructose or meat consumption, and comorbid conditions such as asthma or gastroenteritis. Furthermore, we were unable to evaluate the potential impact of puberty on serum uric acid levels due to the absence of data on the degree of sexual maturity in the KNHANES dataset. This may limit the conclusions previously drawn about the relationship between the onset of puberty and changes in sex hormones and increased SUA levels. Additionally, we cannot exclude the possible relationships between the other variables under investigation and an increase in SUA levels. Further research that considers and controls for other potential causes of an increase in SUA levels is needed to gain a more comprehensive understanding of the factors contributing to MetS. Last, due to our cross-sectional study not ascertaining the mechanisms behind the sex-specific variations in SUA levels and the increased OR for MetS, future research should analyze the changes in SUA levels in conjunction with sex hormone fluctuations and explore the underlying mechanisms using appropriate animal and cellular models.

In conclusion, we demonstrated sex- and age-dependent changes in SUA levels in adolescents based on the updated KNHANES. SUA levels tended to increase with obesity status in both males and females. In addition, the elevation of SUA levels was closely associated with MetS and its components though it exhibited sex-specific differences. Our results showed that the assessment of SUA levels is useful in predicting MetS prevalence and risk, even in nonobese subjects. Therefore, it might be helpful to address elevated SUA levels in a timely manner, as this can significantly reduce the risk of childhood MetS potential cardiometabolic complications in adulthood.

## Data availability statement

The datasets presented in this study can be found in online repositories. The names of the repository/repositories and accession number(s) can be found below: https://knhanes.kdca.go.kr.

## Ethics statement

The studies involving humans were approved by The IRB of Hallym University Chuncheon Sacred Heart Hospital (IRB No. CHUNCHEON 2021-10-004). The studies were conducted in accordance with the local legislation and institutional requirements. The human samples used in this study were acquired from The KNHANES data is a cross-sectional, nationally representative survey that is conducted annually by the Division of Chronic Disease Surveillance, Korean Centers for Disease Control and Prevention (KCDC). Written informed consent for participation was not required from the participants or the participants’ legal guardians/next of kin in accordance with the national legislation and institutional requirements.

## Author contributions

YJ-S designed the study, drafted the manuscript, and analyzed the publicly available data set. HL and JH reviewed and revised the manuscript and provided important intellectual content, including the conceptualization of the study design. YS supervised all aspects of the preparation of the manuscript and assisted with the formulation of the study, analysis of the data, writing of the manuscript, and interpretation of the findings. All authors contributed to the article and approved the submitted version.
